# Assessing the physical environment of older people’s residential care facilities: development of the Swedish version of the Sheffield Care Environment Assessment Matrix (S-SCEAM)

**DOI:** 10.1186/1471-2318-15-3

**Published:** 2015-01-07

**Authors:** Susanna Nordin, Marie Elf, Kevin McKee, Helle Wijk

**Affiliations:** School of Education, Health and Social Studies, Dalarna University, Falun, Sweden; Sahlgrenska Academy, Institute of Health and Care Sciences, Gothenburg University, Gothenburg, Sweden; Karolinska Institute, Department of Neurobiology, Care Sciences and Society, Stockholm, Sweden; School of Architecture, Chalmers University of Technology, Gothenburg, Sweden; Sahlgrenska University Hospital, Gothenburg, Sweden

**Keywords:** Questionnaire, Translation, Validity, Reliability, Health care environment, Residential care facility, Elderly care

## Abstract

**Background:**

There is emerging evidence that the physical environment is important for health, quality of life and care, but there is a lack of valid instruments to assess health care environments. The Sheffield Care Environment Assessment Matrix (SCEAM), developed in the United Kingdom, provides a comprehensive assessment of the physical environment of residential care facilities for older people. This paper reports on the translation and adaptation of SCEAM for use in Swedish residential care facilities for older people, including information on its validity and reliability.

**Methods:**

SCEAM was translated into Swedish and back-translated into English, and assessed for its relevance by experts using content validity index (CVI) together with qualitative data. After modification, the validity assessments were repeated and followed by test-retest and inter-rater reliability tests in six units within a Swedish residential care facility that varied in terms of their environmental characteristics.

**Results:**

Translation and back translation identified linguistic and semantic related issues. The results of the first content validity analysis showed that more than one third of the items had item-CVI (I-CVI) values less than the critical value of 0.78. After modifying the instrument, the second content validation analysis resulted in I-CVI scores above 0.78, the suggested criteria for excellent content validity. Test-retest reliability showed high stability (96% and 95% for two independent raters respectively), and inter-rater reliability demonstrated high levels of agreement (95% and 94% on two separate rating occasions). Kappa values were very good for test-retest (κ = 0.903 and 0.869) and inter-rater reliability (κ = 0.851 and 0.832).

**Conclusions:**

Adapting an instrument to a domestic context is a complex and time-consuming process, requiring an understanding of the culture where the instrument was developed and where it is to be used. A team, including the instrument’s developers, translators, and researchers is necessary to ensure a valid translation and adaption. This study showed preliminary validity and reliability evidence for the Swedish version (S-SCEAM) when used in a Swedish context. Further, we believe that the S-SCEAM has improved compared to the original instrument and suggest that it can be used as a foundation for future developments of the SCEAM model.

**Electronic supplementary material:**

The online version of this article (doi:10.1186/1471-2318-15-3) contains supplementary material, which is available to authorized users.

## Background

The impact of the physical environment on health and wellbeing is well established, and the World Health Organization has conceptualised the physical environment as one important component of quality of life [1]. With regard to the importance of the physical environment for health care, Ulrich et al. [2] in a research review, found evidence that well-designed health care settings can promote healing in patients and provide good working environments for staff according to work satisfaction [2]. Additionally, studies have shown that environmental design is linked to improved well-being and a decrease in psychiatric disturbance among persons with dementia [3]. However, the relationship between the physical environment and behavior in persons with dementia is not yet fully understood, and the research evidence is weak regarding some environmental aspects. For example, several studies have shown that private rooms have positive outcomes on persons with dementia, while the importance of outdoor spaces for this group is less evident. In addition, it is considerable variation regarding the quality of studies in this area, and more well-designed studies are needed [4, 5].

More research is required on how the physical environment of healthcare settings affects health and well-being, so as to fully inform an evidence-based design (EBD) approach, in the planning, designing and construction of health care facilities [6, 7]. As part of this process, reliable and valid instruments are required for the detailed assessment of health care environments. This paper describes an instrument for assessing the physical environment of residential care facilities (RCFs) for older people, the Swedish version of the Sheffield Care Environment Assessment Matrix (S-SCEAM).

Many health care buildings have been designed with a focus on clinical efficiency. Safety issues and infection control have contributed to environments that can be experienced as institutional and impersonal [8]. While RCFs for older people often are represented and promoted as homes rather than health care environments [9], health and safety regulations and the requirements for group living can militate against a homelike atmosphere. The growing influence of person-centred care in RCFs, however, has drawn attention to the importance of the environment for the well-being of residents. Person-centred care emphasizes the personal experience of illness irrespective of age or level of cognitive function [10, 11] and stresses the impact of the physical environment where the care is provided [12–14]. A person-centred environment in RCFs supports privacy and integrity via small-scale settings [4], and promotes a more homelike milieu through open-plan living spaces and private rooms with personal belongings [15]. While attention to health and safety issues will always be necessary in RCFs, there is an argument that facilities that are designed with an intention to balance issues of safety with the goal of supporting a person-centred care approach will result in facilities that most effectively promote both a high quality of care and residents well-being [16, 17].

To be able to determine which outcomes of care are influenced by which specific or combined features of RCF environments is a challenging task. There are also complex relationships to assess, for example between buildings, the building users (staff and residents) and the management of a building [18]. If our understanding of these processes is to be enhanced, reliable and valid instruments for assessing the physical environment in RCFs are required. However, such instruments are lacking, especially instruments that encompass environmental features related to building legislations, health and safety issues, and those related to person-centred care. A recent review of relevant instruments [19] identified one instrument that met several important criteria for having application in the assessment of RCFs, the Sheffield Care Environment Assessment Matrix, SCEAM [20]. SCEAM provides a comprehensive assessment of the physical environment of RCFs. It can be used to guide the process of designing new RCFs; to assess the potential of a building to be used as a RCF; to assess the quality of an RCF in current use; and to collect data for research purposes. SCEAM consists of 370 items, each relating to a specific building feature. These are organised within a series of location categories (e.g.: day spaces, private rooms). Person-centred care is represented in the instrument through the combination of items into user-need domains theorized as central in the occupancy of such buildings, e.g.: privacy, safety and health, cognitive support, and awareness of the outside world. Such user-needs and the items themselves were derived from an exhaustive review of research on RCFs, analysis of building guidelines, and consultation with stakeholders from architecture, building commissioning, providers of health care for older people and RCF residents themselves. When evaluating a building using SCEAM, the assessor walks through the RCF and scores each SCEAM item as present (1) or absent (0) on a checklist. The RCF’s scores is calculated as the proportion of items scored as present, and scores broken down by domain and building location give a profile that can be considered against standards of interest to the assessor or in comparison to other buildings. There is a SCEAM manual with guidelines for use and scoring checklists, and a glossary providing definitions of words and concepts. SCEAM was shown to possess some construct validity, but to date test-retest and inter-rater reliability have not been investigated and the internal consistency of the instrument’s user need domains remain unreported [20].

While SCEAM seems an appropriate instrument for assessing RCFs in the United Kingdom (UK) where the instrument was developed, the present study considered its suitability for use in Swedish RCFs. While British and Swedish RCFs share many features, there are also many differences. Such differences reflect how the two countries diverge on, for example, the specifics of design requirements and contemporary legislation on care for older people; and also more broadly in terms of architectural traditions and the cultural norms governing how older people are perceived and cared for [21–23]. While adapting an instrument designed for one culture so it is fit for use in a different culture is not a simple process [24], there is still more work required in developing an entirely new instrument. Given the lack of instruments for assessing the physical environment in Swedish RCFs, the present study sought to translate, adapt, and further develop SCEAM to make it available for use in Sweden. In order to ensure quality in terms of conceptual and semantic equivalence between the original and the translated instrument, the approach recommended by Polit and Beck [25] was followed [25]. This paper presents results from the translation and adaptation process, providing some initial evidence of the content validity and reliability of the Swedish version of SCEAM, S-SCEAM.

## Method

This study had a mixed-method design, and was conducted in six stages, covering translation and adaptation. The translation phase involved: 1) forward translation and 2) backward-translation. The adaptation phase involved: 3) first test of content validity of the target language instrument; 4) consultation with experts and further adaptation; 5) final test of content validity of the revised target language instrument and 6) reliability tests of test-retest and inter-rater reliability. This procedure was iterative and cyclic with repeated rounds of adjustments performed by the research group.

The study was conducted during 2011–2012, and was a part of a larger project, approved by the Regional Ethical Review Board in Uppsala, Sweden (Ref. No 2011/323). Quantitative data analysis was performed using SPSS for Windows v. 22.0. Participants and materials are described below as they relate to the two phases and six stages.

### Stage 1: Forward translation

Three members of the research group had Swedish as mother tongue (SN, ME, HW), and one was a native English speaker who had been involved in the development of SCEAM (KM). Initially, KM explained and clarified the concepts and meanings of the items to reduce the risk of misinterpretation [26]. The original SCEAM was translated from English to Swedish by the first author (SN), with focus on preserving the meaning of each item. The translation was reviewed and discussed in the research group frequently before reaching consensus on the most appropriate translation of concepts and wording.

### Stage 2: Backward translation

The Swedish version of SCEAM was translated back to English by a bilingual professional translator with English as mother tongue. The translator had no access to the original version. The meaning of the back-translated items and the original items were compared and discussed by the research group and the professional translator before reaching satisfactory equivalence between the versions.

### Stage 3: First test of content validity of the target language instrument

A panel of fourteen Swedish experts (n = 14) with strong professional, personal or research experience on the topic and with profound knowledge about the key construct and the target population were invited. The expert panel was convened through purposive sampling of individuals drawn from construction planning (n = 3), architecture (n = 4), geriatric care (n = 5), and members of senior citizen’s associations (n = 2), in order to gather informed views on the relevance of SCEAM items for use in Swedish RCFs. Thus, the panel represented a mix in terms of roles and disciplines, and were from different geographical locations in Sweden.

The experts were asked to rate all items in terms of relevance on a four-point scale from *not relevant* to *highly relevant*, with an additional response option *do not understand the item*. In addition they were encouraged to comment on the items, the instrument’s form, layout and legibility, and to suggest new items. In line with standard ethical protocol [27], returned completed ratings of the instrument were held to constitute informed consent on the part of the respondent to participate in the study.

The content validity index (CVI) is a method to enhance the construct validity of an instrument, and measures whether the construct is appropriately represented by the items. Item content validity (I-CVI) and scale content validity (S-CVI) were calculated based on the expert ratings [28]. I-CVI was computed for each item by adding together the number of experts rating the item *quite relevant* or *highly relevant*, divided by the total number of experts rating the item. S-CVI was calculated by summing the average I-CVI values and dividing them by the number of items. This approach is recommended when there are many experts involved [28].

### Stage 4: Consultation and further adaptation

In the fourth stage, one or two experts from each profession/background were selected for semi-structured interviews (n = 6). The interview schedule contained open-ended questions designed to obtain the experts’ reflections on the suitability for use of the Swedish version of SCEAM, and how decisions about the relevance of items were made. The interviews lasted between 20 and 50 minutes, were tape-recorded and transcribed verbatim. A content analysis on a manifest level was used to analyse the interviews and also the written comments provided by all the experts in Stage 3. The material was read several times to identify meaning units in line with the aim of the study. The meaning units were condensed and grouped into categories [29]. During the process of adapting the instrument, both quantitative and qualitative data results were considered carefully. All items showing low CVI scores were discussed within the group in the context of the available qualitative data before adjusting, rejecting or adding an item.

### Stage 5: Test of content validity of the revised target language instrument

Polit and Beck [25] argue that a subset of the experts used in the first round of content validation should be carefully selected for the second round of content validation, in order to enhance value [25]. Following the criteria for selection suggested by Polit and Beck, two architects and a nurse (n = 3) were selected due to their competence and commitment during the first round, the consistency of their ratings, and their provision of the most comprehensive feedback. These persons were instructed to rate each item within the revised instrument in terms of its relevance on the four- point scale, as per Stage 3. I-CVIs were calculated again, and an S-CVI calculated as the averaged I-CVIs.

### Stage 6: Reliability tests; test-retest and inter-rater reliability

Six units within a single RCF were selected to test the reliability of the revised SCEAM.

The RCF was selected on pragmatic grounds since the RCF was not under consideration of any building changes during the period of testing the instrument. In addition, the physical environment of the RCF represented a wide range of variation between different floors. Inter-rater reliability was determined by two researchers carrying out the SCEAM assessment independently within the six units during the same day, with no awareness of each other’s scoring, while test-retest reliability was determined by the same researcher assessing the six units twice, with assessments separated by two weeks in accordance with accepted procedure [30].

The two researchers conducting the inter-rater reliability tests had different backgrounds. Rater 1 had extensive experience in nursing research and research in health care settings for older people. In addition, rater 1 had expertise in instrument development, but was not involved in the adaptation process in the present study. Rater 2 had experience in nursing care and was a member of the research team and actively participated in the adaptation process of the instrument. Prior to the inter-rater reliability tests, the two raters discussed all items and practised assessments together. Cohen’s kappa (κ) and consensus estimation were used to determine the level of stability and equivalence [31]. Level of agreement was calculated as the proportion of identical scores when comparing the test results between the two raters and between the first and the second measurement occasion.

## Results

**Figure 1:**
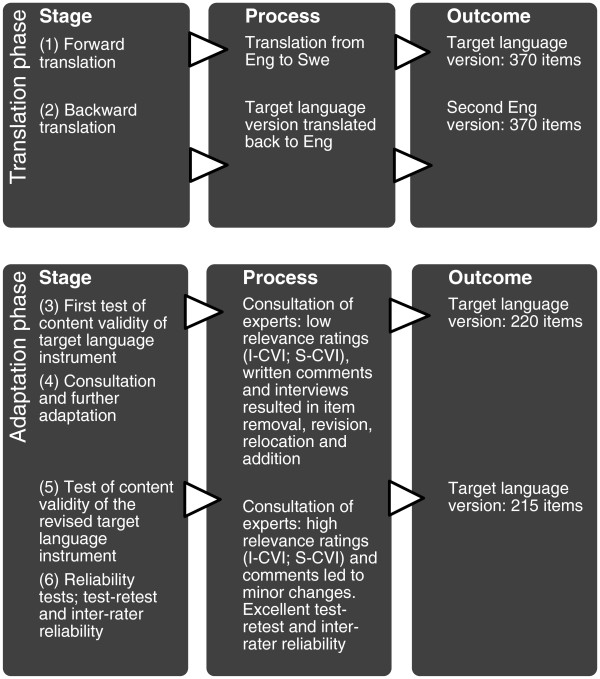
**Flow chart of the process of translation and adaptation.**

The results follow the sequential order in which the translation and adaptation process was performed. Given that the results are drawn from both quantitative and qualitative data, some interpretation of the results is provided in the text, together with reflection on the process itself, in order to provide the results with their appropriate context. A flow chart of the overall process is provided (Figure 1) to demonstrate how the original SCEAM was transformed via item reduction and addition into the Swedish version of SCEAM, S-SCEAM.

Some items in the original SCEAM instrument were found to be difficult to translate. For example, the English word *culture* can be interpreted on several levels and cannot be directly translated to Swedish without the risk of changing the intent of the item. Further, some items in the original version contained words that do not convey the same meaning to Swedes or words that does not exist at all in the Swedish language. For instance, the word *pastiche* is not commonly used in the Swedish language and its meaning was therefore not clear. Furthermore, the phrase *double banked corridor* was not possible to translate since there is no counterpart in the Swedish language. These issues are elaborated via exemplar items in Table 1.

**Table 1:** **Example of forward translation**

Original version	Forward translation
Do the lounges reflect the culture of residents?	Do the lounges generate a feeling of familiarity among the residents?
Is there any pastiche?	Are there any imitations?
Are there mainly double-banked corridors? (rooms on both sides, no windows)	Are there mainly corridors with rooms on both sides, not any windows?

Overall, there was a high degree of consistency between the backward translation and the original version. However, some alterations were revealed between the two versions which led to further investigation of some words and concepts before reaching consensus. Words of similar meaning were discussed such as the English words *safety* and *security*. In the backward translation the word security was used instead of safety and this issue was discussed with the professional translator, where consideration was given to how similar terms can diverge in their suitability for describing a physical state in comparison to describing how a person feels about a physical state. These issues are elaborated via exemplar items in Table 2.

**Table 2:** **Example of backward translation**

Original version	Backward translation
Are there safety lighting indicating paths, ramps, steps?	Is there security lighting to show paths, ramps, steps?

The results of the content validity analysis showed that more than one third of the items had I-CVI values less than the critical value of 0.78 [28], with scores ranging from 0.13 to 0.77. However, there were only a few items receiving very low values, and these were mainly concerning bathroom facilities reflecting diverse standards between the two countries. A majority (75%) had I-CVI scores between 0.51 and 0.77 and these items were estimated to have low relevance in general and were not related to specific domains or location categories.

The analysis of the qualitative data indicated that most of the experts welcomed the underlying idea of assessing the physical environment by means of using an instrument, and some thought that it could be useful when planning new RCFs. However, the overall opinion was that the instrument was too comprehensive and time consuming, and that several items were not relevant in Swedish RCFs, as illustrated in the following quote:

*"It took me a long time to go through the form, I of course wonder if it is possible to shorten it in a future Swedish version. The instrument has a flavour of British old people’s home-culture. Some of the items feel strange but are probably natural in the UK. As I see it, further translation is needed*" (informant 4, architect).

The result of the CVI analysis, with interpretation as aided by the qualitative data, led to the removal and revision of items within the instrument. Nearly one third of the original items were removed (n = 109). Many items from the original SCEAM were found to have low relevance in Sweden due to cultural differences between the residential care systems. Some environmental features do not exist within Swedish RCFs. For example, one item asked whether the personal room or apartment could be monitored by glazed panel, spy-hole or surveillance camera. Such monitoring is not used in Swedish RCFs and is strictly regulated by law (Swedish Code of Statutes 2013:460). On the other hand, some environmental features are universal in Sweden, but not in the UK. For instance, in the original instrument there are several items on personal belongings in the private apartment. That older persons in Swedish RCFs have their own apartments is regulated by the law (Act of renting, Chapter 12 1970:994) which means that they can equip the apartment as they wish:

*"Well, you rent an apartment in a residential care facility just as you rent otherwise bringing your own furniture and all that*" (informant 1, older people´s representative).

This highlights a problem with the original SCEAM, which was developed for use both as a design guide and for post-occupancy evaluation. For use as a design guide, it is important to have building regulations embedded in the instrument as items. However, when the same instrument is used for post-occupancy evaluation, if the RCFs assessed have been constructed according to building regulations as would be anticipated, such items will always return a positive response, creating bias in the instrument’s scoring range, and providing little descriptive information.

The analysis also showed that several items in the original SCEAM were focused on how the building was used, and not on built-in design. One of the experts pointed out the mixture of items assessing different aspects of RCFs such as building design, care organization or work practice, creating the impression of an unfocused instrument. Items related to building orientation, for example asking if lounges or private rooms are facing south in order to get daylight, were also considered to pose difficulties since it is questionable whether it is desirable for all rooms to face the same direction. One of the experts voiced the problem with such items:

*"This is difficult." All windows cannot face south and south is a tricky direction since it can be too hot. If all had been facing north it had been bad, but all facing south is not good either. More important is to see how the light changes during the day, I mean windows in all or several directions"* (informant 9, architect).

Several items were subjective estimations on the level of comfort within the physical environment, e.g. the level of cleanliness. Experts felt that such items would have low reliability, and would also be overly influenced by how the buildings were used. Such concerns resulted in low relevance ratings for these items.

Some items were perceived as complex or difficult to understand due to unclear and ambiguous wording. For instance, some experts objected to the use of the word *homeliness* because of difficulties in defining its meaning and the risk of highly individual interpretations. In addition, items sometimes included more than one sub-query which made the scoring problematic. Some items were perceived to be similar to each other, and some items contributed their respective scores to more than one user-need domain, problematic due to the inflation of inter-domain correlations that results. Consequently, words and concepts were changed and clarified. Overlapping items in which the content was similar or closely related were combined, and items with two or more scoring components were simplified. In cases where a single item scored on multiple domains, the item was revised or partitioned into two items to ensure its score contributed to only one domain. A further 26 items were removed from SCEAM as a result of these revisions and these issues are elaborated via exemplar items in Table 3.

**Table 3:** **Example of revision of items**

Original version	Translated version
*Is there a view of the outside from corridors? (include internal courtyard)*	*Are there external views from different parts of the circulation space? (including internal courtyard)*
*Are there external views from different parts of the circulation space?*	

A number of items had to be allocated to new location categories, as some of the RCF location categories in the original SCEAM had no or little relevance for Swedish RCFs. New items were also added to the target language instrument. Several items relating to building standards and legislation for Swedish RCFs were added and included in the sections ‘Overall Building Layout’ (4), ‘Lounge’ (1), ‘Dining Room’ (1) and ‘Personal Rooms’ (12). For example, accommodations for persons with disabilities are recommended to be free from elevated thresholds in order to improve access in the indoor environment and minimize the risk of falling. The experts also highlighted various environmental aspects of RCFs such as energy use, accessibility and internet access. One informant described her thoughts about modern technology in residential care:

*"I’m thinking, when we become old and get into a residential care facility, we are used to have, to be connected to the internet and mobile phones and all that"* (informant 8, building planner).

The layout of the instrument was also modified in order to obtain greater clarity and structure. The experts felt the layout and format of the instrument created uncertainty regarding which space or room was intended for assessment. Further, several items were repeated in different sections within the instrument, or were perceived to be wrongly located within the instrument. The target language version was therefore modified in order to obtain a more structured layout by clearly separating each section within the form, or reallocating items to location categories for which they were judged most appropriate. In the ‘Personal Rooms’ category, *Bathroom* was included as a separate sub-category due to specific requirements within the Swedish construction standards. Finally, some items in the original SCEAM were negatively worded (n = 44), and their I-CVI scores indicated that they were regarded as of low relevance. Therefore, all items were reworded positively (see Table 4 for example items).

**Table 4:** **Example of a negatively worded item**

Original version	Translated version
*Are there any intrusive safety/security devices?*	*Are safety/security devices discretely integrated in the environment?*

The relevance of the revised set of items was again submitted to expert scrutiny. The written comments from the experts reflected on new issues that had not come to light when the previous version of SCEAM had been considered. One of these aspects concerned the possibility to share an apartment as a couple, which was emphasized by one of the experts as an important aspect of the quality of life of older persons in residential care. Another important suggestion was that RCFs should have environmental features that provide residents with the option to control the amount of daylight via blinds or curtains. As a result of the experts’ input, 3 new items were added while 8 items were removed, of which 2 were felt to duplicate existing items. Two items were moved from the ‘External/Entrance’ location category to the ‘Overall Building Layout’ location category. These changes resulted in a final version of the adapted SCEAM (S-SCEAM) containing 215 items (Additional file 1). Tables 5 and 6 presents a matrix of the number of items in the original SCEAM and in S-SCEAM by location category and user-need domain.

**Table 5:** **Original version: number of items by location category and domain**

Single domains	External/entrance	Lounge	Dining room	Bathroom	WC	Personal rooms	Overall layout	Staff	Garden	Plans items	Sum
Safety/health	5	6	6	8	9	11	11	-	4	1	**61**
Physical support	3	4	2	13	7	8	13	-	6	2	**58**
Cognitive support	-	1	-	-	-	2	21	-	1	-	**25**
Normalness/authenticity	1	10	5	4	1	5	5	-	2	-	**33**
Personalisation	-	3	-	1	-	11	5	-	1	-	**21**
Choice/control	-	1	1	2	-	6	10	-	3	-	**23**
Privacy	3	4	1	9	6	13	6	-	1	2	**45**
Comfort of the environment	-	8	2	2	2	9	8	-	2	-	**33**
Awareness of outside world	4	6	-	-	-	5	10	-	5	2	**32**
Community	11	1	-	-	-	-	6	-	-	2	**20**
Staff	-	-	-	-		-	-	7	-	-	**7**
**Multiple domains**											
Safety/health Choice/control	1										**1**
Safety/health Comfort of the environment		1	1	1	1	1	1				**6**
Personalisation Safety/health						1					**1**
Physical support Privacy							1				**1**
Choice/control Cognitive support									1		**1**
Choice/control Privacy										1	**1**
Awareness of outside world Personalisation Physical support										1	**1**
**Sum**	**28**	**45**	**18**	**40**	**26**	**72**	**97**	**7**	**26**	**11**	**370**

**Table 6:** **Swedish version: number of items by location category and domain**

Domains	External/entrance	Lounge	Dining room	Bathroom	Personal rooms	Overall layout	Garden	Sum
Safety/health	4	3	2	5	11	8	2	**35**
Physical support	3	3	3	6	12	13	4	**44**
Cognitive support	-	1	-	-	3	11	1	**16**
Normalness/authenticity	1	4	4	2	2	3	1	**17**
Personalisation	-	1	-	-	1	3	1	**6**
Choice/control	-	1	1	-	4	12	3	**21**
Privacy	-	3	1	5	5	6	1	**21**
Comfort of the environment	-	2	2	1	5	1	1	**12**
Awareness of outside world	4	3	-	-	6	7	4	**24**
Community	10	1	-	-	2	6	-	**19**
**Sum**	**22**	**22**	**13**	**19**	**51**	**70**	**18**	**215**

A second content validation analysis was carried out on the revised set of items, with I-CVI scores above 0.78 and S-CVI score above 0.90, the suggested criteria for excellent content validity [25]. The test-retest reliability analyses for S-SCEAM showed a stability of 96% for rater 1 and 95% for rater 2. The agreements between the first and the second assessments were κ = 0.903 (95% CI [0.85, 0.95]) and κ = 0.869 (95% CI [0.81, 0.93]) respectively for rater 1 and rater 2. Regarding inter-rater reliability, analysis of the data from the first assessment indicated 95% agreement between the two raters (κ = 0.851 (95% CI [0.79, 0.91]), while the second assessment demonstrated 94% agreement between the two raters (κ = 0.832 (95% CI [0.76, 0.90]). Of the individual items scored differently by the two raters, most were found in the ‘Overall Building Layout’ location category, while the highest proportion of items with agreement was found in the ‘Dining room’ location category. In terms of the instrument’s user-need domains, at the first measurement occasion, the domain ‘Personalisation’ had the lowest inter-rater reliability (κ = 0.154), and the domain ‘Awareness of the outside world’ had the lowest inter-rater reliability at the second measurement occasion (κ =0.444). The highest inter-rater reliability was found in the domain ‘Comfort’ at both measurement occasions (κ =1.000). These results indicate that overall S-SCEAM had excellent test-retest and inter-rater reliability [31, 32].

## Discussion

### Main findings

Our procedure for the translation, adaptation and development of SCEAM from English into Swedish produced an instrument, S-SCEAM, with excellent test-retest and inter-rater reliability, good face validity and excellent content validity. Following the initial translation, our careful review of the instrument via expert consultation led to the removal, reallocation, revision or addition of a number of items. Wholesale changes to an original instrument raises the issue of whether or not one has produced an adaptation of an existing instrument, or rather a new instrument entirely. Yet, the basic structure of the original instrument remains, as do a high proportion of original items (n = 90%). Importantly, the scoring system, based on the relationship between environmental features on the one hand and resident/user needs on the other - the manifestation of the person-centred care philosophy on which SCEAM is founded – is retained. An argument can be made that subtracting or adding items to an instrument is only changing one level of that instrument: the original SCEAM instrument was not merely a series of items assessing some abstract idea of a ‘good’ care environment for older people, but rather a tool for examining the role of the physical environment in the quality of life of its residents, and for enhancing the positive influence of that environment [33]. As long as the adaptation process from original to new instrument is carefully and methodically performed, with sensitivity to the original instrument’s purpose and theoretical foundation, the core instrument remains the same.

### Cultural adaptation of instruments

The fact that SCEAM had been developed in a British health care context suggested it might be suitable for adaptation for use in Sweden. Swedish and British citizens share some core values [22], and the two countries are both subjected to European legislation and have long histories of welfare provision in supporting disadvantage citizens such as old people [34, 35]. Nevertheless, care facilities in the two countries could be anticipated to be distinct in terms of care practices, differing planning procedures and building legislations, differences in the materials used in the respective construction industries, and different principles relating to exterior and interior design [36, 37].

This distinctiveness of Swedish and British RCFs emerged quite clearly in this study. The first analysis of the content validity of the translated SCEAM showed many items to perform poorly, with the instrument overall having an unacceptable S-CVI score. Core differences between Swedish and British RCFs – such as the requirement for individual private apartments in the former, informed by Swedish legislation identifying care facilities as ‘home’ for their residents [9] - had the effect that many items in the original SCEAM were judged to have little or no relevance for the Swedish context. Of course, not all the changes in the adaptation process were the result of differences between care cultures. Some issues had nothing to do with the instrument’s application, for example problems with item construction and instrument layout. The passage of time itself can also influence the validity of an instrument. The ‘shelf life’ of an instrument will vary depending upon its area of application, and researchers who adapt existing instruments for new contexts need to be sensitive to how such new contexts can differ from the original development context on many dimensions [38, 39]. Although SCEAM was developed only a decade ago, ten years is a substantial duration in which developments and changes in care practices and legislation can occur, as well as in technology relevant to both care practice and care environments. Adapting an instrument from one language and culture into another is, therefore, not only about ensuring cultural relevance and applicability: the adaptation process provides an opportunity to look carefully at the original instrument and to improve upon the original in terms of reliability and validity, general robustness and ‘user-friendliness’, and suitability for use.

### Study strengths and weaknesses

Due to the breadth of the original SCEAM instrument, the translation and adaptation work was time consuming and demanding. However, it was crucial that the process was comprehensive, and the iterative approach adopted provided opportunities for periods of reflection, adaptation and revision. Several studies have emphasized the importance of taking into account contextual factors in order to adapt and validate instruments developed in one country so that they can be used in another [40, 41]. Eldh et al. [42] stresses the necessity of forward- and backward translation of an instrument followed by testing the translated instrument in the target culture, in order to achieve a better understanding of important aspects of both the original and translated version [42]. It was valuable in this respect to have one of the members of the group that developed the original SCEAM as part of the current research group; and the input of a range of individuals representing different perspectives and different expertise was essential.

To ensure quality in terms of conceptual and semantic equivalence between the original and the translated instrument, a forward-backward translation recommended by Polit and Beck [25] was followed in the current study [25]. However, standard guidelines for instrument translation and adaptation are lacking, and there is no accepted optimal method. Most authorities on this topic suggest the use of multiple methods [26, 43], an advantage of which is that limitations in one method can be compensated by another [44]. For example, CVI analysis focuses on the items at hand and does not elucidate other issues that might be of importance to adequately measure the underlying construct [28]. The CVI values served as a basis for further analysis and discussion in the research group but were not the sole criteria for item removal or transformation. In our study we collected qualitative data in addition to performing CVI analysis, so that we could more easily interpret the reasons why items were performing poorly, and this helped us to improve items and to develop new items.

The test-retest and inter-rater reliability data analysed in the present study were collected from a single RCF, which could be regarded as problematic. However, the facility contained six units that varied in layout and environmental features, so it is unlikely that the reliability estimates were inflated due to insufficient environmental variation. Further validation analyses for the new instrument cannot be undertaken until data has been acquired from a range of facilities, a task out with the remit of the present study. As an example, the internal consistency and conceptual underpinning of the user-need domains in S-SCEAM requires further examination, while the feasibility and suitability for use of S-SCEAM will only be confirmed following extended use.

## Conclusions

There is a lack of instruments that reliably and validly assess the physical environment of RCFs, yet there is a great need for such instruments. There is clear evidence for the important role of the physical environment in the quality of life of individuals, and as an individual’s frailty increases so does the significance of the physical environment for life quality [45]. Of course, physical environments, once constructed, are not so easily changed. Yet the cost of intervention at a physical level is not an argument against so intervening, and the retention of a poor environment can be a serious barrier to the success of interventions other than environmental: if the environment works against the provision of good quality care, the impact of change in care practice will be minimised.

S-SCEAM can contribute to the evidence-based design of RCFs through use when planning new facilities, assessing existing facilities, renovating facilities, or when conducting esearch into the relationship between the environment of RCFs and outcomes such as quality of life of residents and the quality of care received, and work satisfaction for staff. In addition, scores on the instrument can be used to make structured comparisons between different residential care facilities in terms of overall environmental quality, or in terms of profiles of scores on domains or within specific building locations, and for the setting of quality targets. In removing items from the original SCEAM that were anticipated to have no or low variation in scoring across facilities, the description of environmental features required by legislation has also consequently been removed. Thus, S-SCEAM cannot in itself be used as a design guide, but rather as an instrument to support the design process. Similarly, the removal of items that relate to how the building is used, as opposed to built, means that a profile of scores comparing ‘built’ with ‘in use’ quality cannot be obtained. In focusing down the purpose of S-SCEAM relative to SCEAM, we believe we have enhanced the performance of the instrument within its remaining areas of use.

We offer S-SCEAM as a comprehensive instrument for the assessment of Swedish RCFs. While further psychometric testing of the instrument is desirable, it possesses good reliability, good face validity and good content validity. Guidelines for use appropriate for the Swedish context are also yet to be developed. Nevertheless, S-SCEAM in its current form has considerable promise. While S-SCEAM clearly has its greatest application within Swedish RCFs, we would suggest that S-SCEAM represents an improvement on the original SCEAM, and can be better used as a foundation for future developments of the SCEAM assessment model.

## Electronic supplementary material

Additional file 1: **Contains the current version of the S-SCEAM checklist.** (PDF 310 KB)
