# Supplementary material for: Assessing the physical environment of older people’s residential care facilities: development of the Swedish version of the Sheffield Care Environment Assessment Matrix (S-SCEAM)
Source: BMC Geriatr. 2015 Jan 7;15:3. doi: 10.1186/1471-2318-15-3 (PMC4323237; doi:10.1186/1471-2318-15-3)
Supplement: Supplementary file 1 — Additional file 1: Contains the current version of the S-SCEAM checklist. (PDF 310 KB) [file 12877_2014_1082_MOESM1_ESM.pdf]

S-SCEAM should not be administered without consulting the guidelines for use. Guidelines for use and information on scoring procedures can be obtained from the first author by request.

S-SCEAM

Äldreboende: ____________________________ Datum:______________

Bedömare: ____________________________

**DELFRÅGA**	**Ja**	**Nej**	**Ej tillämpligt**

Externt/entré

1. Är äldreboendet integrerat i ett vanligt bostadsområde?	○	○	○
2. Finns det serviceinrättningar och butiker i närheten?	○	○	○
3. Ligger äldreboendet i närheten av kollektivtrafik?	○	○	○
4. Ligger äldreboendet i ett område där människor rör sig dagligen?	○	○	○
5. Finns det natur eller trädgård i närheten?	○	○	○
6. Finns det besöksparkering?	○	○	○
7. Finns det reserverad parkering för rörelsehindrade inom 25 meters gångavstånd från huvudentrén?	○	○	○
8. Är gångvägen från bilparkeringen säker? (så att boende eller besökare inte behöver korsa parkeringen där bilar är i rörelse)	○	○	○
9. Är det säkert att nå byggnaden till fots? (säkra övergångsställen, gångväg som är avskild från bilväg)	○	○	○

Externt/entré

10. Finns det belysning som anger stigar, ramper, trappsteg?	○	○	○
11. Finns det rörelsestyrd belysning som tänds då någon närmar sig byggnaden?	○	○	○
12. Är huvudentrén utformad så att den är lätt att hitta?	○	○	○
13. Finns det någon belysning nära huvudentrén?	○	○	○
14. Är huvudentrén klimat- och väderskyddad?	○	○	○
15. Finns det klimat- och väderskyddade sittplatser utomhus i direkt anslutning till huvudentrén?	○	○	○
16. Är huvudentrén utformad så att det är möjligt att lämna/hämta personer eller personliga tillhörigheter?	○	○	○
17. Är huvudentrén utformad för personer med nedsatt rörelse- eller orienteringsförmåga?	○	○	○
18. Finns det ringklocka, porttelefon eller instruktioner om hur man kommer in för att välkomna besökare?	○	○	○
19. Finns det minst två sittplatser innanför huvudentrén?	○	○	○
20. Finns det någon reception i huvudentrén?	○	○	○
21. Finns det informationstavlor för besökare?	○	○	○
22. Är trygghets/säkerhetsanordningar diskret integrerade i miljön? (t ex störande anslagstavlor, blinkande larm, kala lysrör)	○	○	○

Vardagsrum

23. Kan man överblicka hela vardagsrummet?	◯	◯	◯
24. Kan personer som har andra ärenden än att besöka anhöriga, nå utrymmen utan att behöva passera igenom vardagsrummet? (t ex fastighetsskötare, brevbärare, studenter)	◯	◯	◯
25. Kan boende och personal nå andra utrymmen utan att behöva passera igenom vardagsrummet?	◯	◯	◯
26. Kan man välja mellan att vara i olika vardagsrum eller liknande rum? (t ex alkov)	◯	◯	◯
27. Är trygghets/säkerhetsanordningar diskret integrerade i miljön? (t ex störande anslagstavlor, blinkande larm, kala lysrör)	◯	◯	◯
28. Är vardagsrummet utformat så att det ger en karaktär av bostad och hem? (material, storlek, form)	◯	◯	◯
29. Är ljussättningen i vardagsrummet utformad på ett sätt som ger en karaktär av bostad och hem?	◯	◯	◯
30. Är sittplatserna utformade på ett sätt som ger en karaktär av bostad och hem? (medicinteknisk utrustning och konstgjorda material är diskreta)	◯	◯	◯
31. Finns det möjlighet att välja typ och höjd på stolar och soffor?	◯	◯	◯
32. Är vardagsrummet utformat så att det finns plats för personliga föremål? (t ex föremål på vägghyllor, tavlor, speglar, golvföremål)	◯	◯	◯
33. Finns det en variation av material som stimulerar de boende? (t ex trä, tyg)	◯	◯	◯
34. Är sittplatserna säkra och stödjande för sköra äldre personer? (t ex inga vassa ytor, stabila armstöd, robusta ben, låg sitthöjd)	◯	◯	◯
35. Är golven halksäkra? (viss strävhet, jämn yta utan springor, material med liknande friktion intill varandra)	◯	◯	◯

Vardagsrum

36. Är vardagsrummet säkert utformat för sköra äldre personer? (t ex skydd på varma/vassa/glasade ytor, en golvnivå eller tydlig övergång från en nivå till en annan, fönster med fönsterlås)	○	○	○
37. Är vardagsrummet tillgängligt för personer med funktionsnedsättningar?	○	○	○
38. Finns det tillräckligt med plats i vardagsrummet för att manövrera en rullstol med hjälpare?	○	○	○
39. Kan de boende ha utblick över natur eller bostadsområde från vardagsrummet? (även personer som sitter i rullstol)	○	○	○
40. Är det möjligt att se vardagliga inomhusaktiviteter inomhus från vardagsrummet? (t ex personal som förbereder mat eller andra hushållssysslor)	○	○	○
41. Finns åtkomst till internet i vardagsrummet?	○	○	○
42. Har vardagsrummet god akustik och ljuddämpning som främjar samvaro, lugn och ro?	○	○	○
43. Är det god ventilation i vardagsrummet?	○	○	○
44. Finns det tillräckligt med dagsljusinsläpp i vardagsrummet?	○	○	○

Kök/matplats

45. Finns det en separat matplats för de boende?	○	○	○
46. Kan personer som har andra ärenden än att besöka anhöriga, nå utrymmen utan att behöva passera igenom köket/matplatsen? (t ex fastighetsskötare, brevbärare, studenter)	○	○	○
47. Är trygghets/säkerhetsanordningar diskret integrerade i miljön? (t ex störande anslagstavlor, blinkande larm, kala lysrör)	○	○	○
48. Är köket/matplatsen utformad så att den ger en karaktär av bostad och hem? (material, storlek, form)	○	○	○

Kök/matplats

49. Är golven halksäkra? (viss strävhet, jämn yta utan springor, material med liknande friktion intill varandra)	○	○	○
50. Är sittplatserna säkra och stödjande för sköra äldre personer? (t ex inga vassa ytor, stabila armstöd, robusta ben, låg sitthöjd)	○	○	○
51. Är köket/matplatsen säkert utformad för sköra äldre personer? (t ex skydd på varma/vassa/glasade ytor, en golvnivå eller tydlig övergång från en nivå till en annan, fönster med fönsterlås)	○	○	○
52. Är köket/matplatsen tillgänglig för personer med funktionsnedsättningar?	○	○	○
53. Finns det tillräckligt med plats i kök/matplats att manövrera en rullstol med hjälpare?	○	○	○
54. Kan alla boende inom boendeenheten äta samtidigt?	○	○	○
55. Är matborden utformade så att de bidrar till social samvaro? (ca max sex personer per bord)	○	○	○
56. Är det god ventilation i kök/matplats?	○	○	○
57. Är köket/matplatsen trivsamt upplyst? (t ex dagsljus eller belysning som är god utan att bländа)	○	○	○

Allmänt badrum

58. Är badrummet placerat så att användaren kan vara privat med tanke på andra boende som rör sig inom byggnaden?	○	○	○
59. Kan man vara privat utan risk för lyhördhet?	○	○	○
60. Är badrummet insynsskyddat?	○	○	○

Allmänt badrum

61. Finns möjlighet till avskildhet inne i badrummet? (alkov, skärm, duschdraperi)	○	○	○
62. Är badrummet utformat så att det ger en karaktär av bostad och hem? (t ex medicinteknisk utrustning och anpassning för funktionsnedsättning är diskret)	○	○	○
63. Finns öppningsbara WC-lås till badrummet?	○	○	○
64. Finns det larmanordning i det allmänna badrummet?	○	○	○
65. Är golvet halksäkert? (viss strävhet, jämn yta utan springor, material med liknande friktion intill varandra)	○	○	○
66. Är badrummet säkert utformat för sköra äldre personer? (t ex skydd på varma/vassa/glasade ytor, en golvnivå eller tydlig övergång från en nivå till en annan, inga fönster eller fönster med fönsterlås)	○	○	○
67. Finns automatisk dörröppnare eller automatisk skjutdörr? (automatisk dörröppnare bör vara försedd med markering av dörrens svepyta alternativt säkerhetssensor)	○	○	○
68. Är det allmänna badrummet utformat så att det kan användas av personer med funktionsnedsättningar?	○	○	○
69. Finns det fritt utrymme vid sidan av dörren så att personer i rullstol kan öppna badrumsdörren utan problem?	○	○	○
70. Finns det tillräckligt med plats i det allmänna badrummet för lyftanordning eller att manövrera en rullstol med hjälpare?	○	○	○
71. Är det tydliga kontraster mellan badrumsinredningen och dess bakgrund?	○	○	○
72. Finns uttag för rakapparat inom räckhåll från rullstol? (ej högre än 1,1 m samt ej placerat i hörn)	○	○	○
73. Finns det tillgång till hygienartiklar för att garantera handhygien? (t ex tvålpumpar, handsprit, handtork/handdukar?)	○	○	○

Allmänt badrum

74. Finns det speglar?	○	○	○
75. Är det god ventilation i badrummet?	○	○	○
76. Är badrummet trivsamt upplyst? (t ex dagsljus eller god belysning som inte bländar)	○	○	○

Enskilda lägenheter

77. Finns det ringklocka eller dylikt vid entrén/dörren?	○	○	○
78. Är entrédörren utformad med tydliga kontraster?	○	○	○
79. Är lås och handtag placerade och utformade så att de kan användas av personer med nedsatt rörelse- och orienteringsförmåga?	○	○	○
80. Finns det tillräckligt med plats i entrén/hallen att manövrera en rullstol med hjälpare?	○	○	○
81. Kan kapphyllan anpassas både för personer som står och sitter?	○	○	○
82. Kan den boende vara privat i sin lägenhet utan risk för lyhördhet? (beakta intilliggande rum)	○	○	○
83. Är ljudmiljön behaglig inne i lägenheten? (så att det går att utestänga ljud från t ex korridor intilliggande rum eller utomhusmiljön)	○	○	○
84. Är trygghets/säkerhetsanordningar diskret integrerade i miljön? (t ex störande anslagstavlor, blinkande larm, kala lysrör)	○	○	○
85. Är lägenheten utformad så att den boende kan placera personliga föremål? (t ex föremål på vägghyllor, tavlor, speglar, golvföremål)	○	○	○
86. Finns det uttag för TV?	○	○	○
87. Finns det telefonjack i lägenheten?	○	○	○

Enskilda lägenheter

88. Finns åtkomst till internet i lägenheten?	○	○	○
89. Finns det något värdeskåp i lägenheten?	○	○	○
90. Kan den boende se ut mot natur eller bostadsområde från lägenheten?	○	○	○
91. Kan personer som sitter i rullstol se ut genom lägenhetens fönster? (fönsterbröstning max 0,80 m över golv)	○	○	○
92. Kan den boende se ut genom fönstret från sängen?	○	○	○
93. Finns det tillräckligt med dagsljusinsläpp i den enskilda lägenheten?	○	○	○
94. Finns anordningar för att kontrollera dagsljuset i lägenheten? (t ex persienner, markiser, gardiner)	○	○	○
95. Kan den boende själv reglera belysningen? (inkluderat person med funktionsnedsättning)	○	○	○
96. Är radiatorer och värmereglage placerade och utformade så att den boende själv kan reglera värmen? (inkluderat person med funktionsnedsättning)	○	○	○
97. Kan den boende själv använda luftkonditionering eller vädra? (inkluderat person med funktionsnedsättning)	○	○	○
98. Är det god ventilation i den enskilda lägenheten utan att man behöver öppna fönster och vädra?	○	○	○
99. Finns det utrymme för samvaro och måltider i lägenheten?	○	○	○
100. Är köket utrustat med köksinredning? (kylskåp, skåputrustning, kokplatta och diskbänk, plats för kaffebryggare) Skatta 1=ja om det finns åtminstone kylskåp och plats för kaffebryggare el. vattenkokare	○	○	○
101. Är köket/pentryt utformat så att det inbjuder till gemensamma aktiviteter?	○	○	○

Enskilda lägenheter

102. Är köket/pentryt utformat med arbets- och förvaringsytor i olika höjder samt plats för mer än en person?	○	○	○
103. Finns det tillräckligt med plats i lägenheten att manövrera en rullstol med hjälpare?	○	○	○
104. Är lägenheten fri från trösklar invändigt?	○	○	○
105. Är golven halksäkra? (viss strävhet, jämn yta utan springor, material med liknande friktion intill varandra)	○	○	○
106. Är lägenheten säkert utformad för sköra äldre personer? (t ex skydd på varma/vassa/glasade ytor, en golvnivå eller tydlig övergång från en nivå till en annan, fönster med fönsterlås)	○	○	○
107. Finns innerdörrar i kontrasterande färg?	○	○	○
108. Är det lätt för den boende att hitta till hygienrummet från sin säng?	○	○	○
109. Finns utrymme för sängbord intill sängen?	○	○	○
110. Är sovrummet mörkt på natten?	○	○	○
111. Finns det någon nattbelysning eller sänglampa?	○	○	○
112. Finns det larmanordning i lägenheten?	○	○	○
113. Är larmsystemet utformat så att det inte är störande inne i lägenheten nattetid?	○	○	○
114. Finns det fritt utrymme vid sidan av dörren så att personer i rullstol kan öppna hygienrumsdörren utan problem?	○	○	○
115. Är dörren till hygienrummet utåtgående eller finns skjutdörr som går in i väggen?	○	○	○
116. Finns öppningsbara WC-lås till hygienrummet?	○	○	○

Enskilda lägenheter

117. Finns det larmanordning i hygienrummet?	○	○	○
118. Är hygienrummet säkert utformat för sköra äldre personer? (t ex skydd på varma/vassa/glasade ytor, en golvnivå eller tydlig övergång från en nivå till en annan, fönster med fönsterlås)	○	○	○
119. Är golvet halksäkert? (viss strävhet, jämn yta utan springor, material med liknande friktion intill varandra)	○	○	○
120. Är badrumsgolvet utformat så att det kan användas av de boende utan risk för att vatten rinner ut från duschplatsen?	○	○	○
121. Är hygienrummet utformat efter den boendes behov? (t ex plats för eventuella hjälpmedel, utrymme för hjälpare)	○	○	○
122. Uppfyller stödfunktioner i hygienrummet den boendes behov? (t ex stödhandtag, armstöd, taklyft, anordningar för att justera höjd)	○	○	○
123. Finns det tillgång till hygienartiklar för att garantera handhygien (t ex tvålpumpar, handsprit, handtork/handdukar?)	○	○	○
124. Finns uttag för rakapparat inom räckhåll från rullstol? (ej högre än 1,1 m samt ej placerat i hörn)	○	○	○
125. Är toalettpappersrullen placerad så att den lätt kan ses och nås från toalettstolen?	○	○	○
126. Finns förvaringsutrymme i eller i anslutning till hygienrummet för hygien- och förbrukningsartiklar?	○	○	○
127. Är det god ventilation i hygienrummet?	○	○	○

Övergripande byggnadsutformning

128. Är äldreboendet utformat så att det är lätt att hitta?	○	○	○
129. Är äldreboendet tillgängligt med tanke på avstånd och hinder?	○	○	○
130. Är äldreboendet utformat så att det påminner om ett vanligt bostadshus?	○	○	○

Övergripande byggnadsutformning

131. Är alla enskilda lägenheter tillräckligt skyddade från insyn av passerande?	◯	◯	◯
132. Är alla vardagsrum tillräckligt skyddade från insyn av passerande?	◯	◯	◯
133. Är boendet utformat så att de flesta enskilda lägenheter får in maximalt med dagsljus?	◯	◯	◯
134. Har alla egen lägenhet?	◯	◯	◯
135. Finns det möjlighet att utforma lägenheten så att två personer kan ha delat boende om de så önskar? (t ex äkta makar)	◯	◯	◯
136. Har man använt naturliga och hållbara material i byggnaden?	◯	◯	◯
137. Är det en effektiv energianvändning i byggnaden med tanke på temperatur och luftkvalitet?	◯	◯	◯
138. Har de boende tillgång till källsortering?	◯	◯	◯
139. Finns det någon besökstoalett som är utformad för personer med olika funktionsnedsättningar?	◯	◯	◯
140. Finns något väder- och klimatskyddat utrymme utomhus avsett för rökning?	◯	◯	◯
141. Finns det trygghetsboende inom eller intill äldreboendet?	◯	◯	◯
142. Kan de boende fritt och obehindrat röra sig i byggnaden? (t ex användbara hissar på lämpliga platser, tillräckligt breda korridorer)	◯	◯	◯
143. Finns det något utrymme som är lättillgängligt och tillräckligt stort så att boende från alla enheter kan samlas? (inklusive hjälpmedel)	◯	◯	◯
144. Finns det utrymme för mindre sammankomster? (t ex familjeträffar, högtider)	◯	◯	◯
145. Finns det något övernattningsrum för besökare?	◯	◯	◯

Övergripande byggnadsutformning

146. Finns det något utrymme för fysisk aktivitet?	○	○	○
147. Finns det något utrymme för fritidssysselsättning eller sociala aktiviteter?	○	○	○
148. Finns det tillträde till dagcentral eller andra aktiviteter inom byggnaden för utomstående?	○	○	○
149. Kan utomstående nå dagcentral eller andra aktiviteter utan att behöva röra sig inom byggnaden?	○	○	○
150. Finns det tillgång till bord eller skrivbord för fritidssysslor någonstans i boendeenheten? (matbord räknas inte)	○	○	○
151. Finns utblickar från olika delar av kommunikationsutrymmena? (inklusive utsikt över innergård)	○	○	○
152. Finns det någon referenspunkt i den yttre miljön? (t ex väg, närliggande byggnad, höga träd)	○	○	○
153. Är korridorerna utformade så att de boende kan ta sig fram utan att komma till en återvändsgränd?	○	○	○
154. Finns det någon gångslinga inom boendeenheten?	○	○	○
155. Är kommunikationsutrymmena utformade så att det finns flera olika sittplatser att välja mellan beroende på vad man tänker göra? (t ex läsa tidningen, se ut över naturen)	○	○	○
156. Är boendeenheten utformad så att de boende lättare kan hitta med hjälp av form, ljus eller färg? (t ex tydlig skyltning, färgkodning)	○	○	○
157. Är de gemensamma utrymmena utformade så att de känns igen utifrån användning?	○	○	○
158. Är vägen mellan de enskilda lägenheterna och de allmänna utrymmena såsom vardagsrum, kök/matplats samt korridorer tydligt markerad genom skyltar eller färgkodning?	○	○	○

Övergripande byggnadsutformning

159. Är lägenhetsdörrarna markerade genom avvikande utformning? (t ex annat material)	○	○	○
160. Är det färre än tre knutpunkter från den lägenhet som ligger längst bort, till vardagsrummet? (t ex korsning i kommunikationsutrymmen som den boende måste ta ställning till)	○	○	○
161. Är det kortare än 15 meter till vardagsrummet från den lägenhet som ligger längst bort?	○	○	○
162. Finns det sittplatser på vägen mellan lägenheterna och vardagsrummet?	○	○	○
163. Ligger lägenheterna åtskilda från allmänna transportstråk?	○	○	○
164. Kan man röra sig överallt inom äldreboendet utan att behöva gå i trappor, alternativt trappor med max 0,15 m steghöjd och minst 0,26 m stegdjup?	○	○	○
165. Finns det ledstänger i kommunikationsutrymmena?	○	○	○
166. Är det tydliga kontraster mellan exempelvis ledstänger, dörrvred och bakgrund?	○	○	○
167. Är korridorerna tillräckligt breda så att två personer i rullstol kan mötas? (minst 1,60 m)	○	○	○
168. Är det minst 80 cm fritt passagemått i dörrar?	○	○	○
169. Är dörrhandtagen utformade så att de kan användas av personer med nedsatt rörelse- och orienteringsförmåga eller nedsatt styrke- grip- eller precisionsförmåga?	○	○	○
170. Är ledstängerna säkert utformade för sköra äldre personer? (runda eller ovala så att det inte finns risk att fastna)	○	○	○
171. Finns det kontrasterande ränder på första och sista steget i trappor samt på ramper och upphöjda trösklar?	○	○	○

Övergripande byggnadsutformning

172. Är utrymningsvägar väl utformade med tydliga skyltar för att underlätta utrymning?	◯	◯	◯
173. Är byggnaden utformad så att det går att följa förändringar av dagsljuset under dygnet?	◯	◯	◯
174. Varierar belysningen mellan rummen på boendeenheten?	◯	◯	◯
175. Varierar temperaturen mellan rummen på boendeenheten?	◯	◯	◯
176. Finns det tillgång till telefon för personer med funktionsnedsättningar? (t ex personer i rullstol, personer med nedsatt arm- och handfunktion) (personalens telefon räknas inte)	◯	◯	◯
177. Finns det någon hörslinga?	◯	◯	◯
178. Har telefonerna inbyggd volymkontroll samt teleslinga?	◯	◯	◯
179. Finns det tillgång till telefon i avskildhet? (personalens telefon räknas inte)	◯	◯	◯
180. Varierar temperaturen under dygnet?	◯	◯	◯
181. Är korridorerna dagsljusbelysta via fönster eller takfönster?	◯	◯	◯
182. Är det särskilt upplyst vid hörn, passager eller andra viktiga funktioner? (avsiktligt starkare ljus från fönster och/eller elektrisk belysning)	◯	◯	◯
183. Kan vårdpersonal ha överblick över allmänna utrymmen såsom vardagsrum, kök/matplats samt korridorer utan svårighet?	◯	◯	◯
184. Finns det larmanordning i korridorerna? (platser där personal kan tillkalla hjälp genom hela kommunikationsutrymmet)	◯	◯	◯
185. Är golven halksäkra i kommunikationsutrymmen? (viss strävhet, jämn yta utan springor, material med liknande friktion intill varandra)	◯	◯	◯

Övergripande byggnadsutformning

186. Är radiatorer skyddade eller inställda på maxtemperatur för att minimera risk för brännskada?	○	○	○
187. Går det att kontrollera vattentemperaturen med termostatblandare?	○	○	○
188. Finns det möjlighet för de boende att själva tvätta kläder om de så önskar?	○	○	○
189. Finns någon plats inom byggnaden där de boende kan köpa kioskvaror som godis, läsk, glass eller toalettartiklar?	○	○	○
190. Kan de boende brygga kaffe/té i de allmänna utrymmena?	○	○	○
191. Finns det ett badrum tillgängligt inom byggnaden så att de boende kan ta ett bad om de så önskar? (med eller utan assistans)	○	○	○
192. Finns det någon snoezelen? (multi-sensoriskt rum med ljus, ljud, mjuka ytor)	○	○	○
193. Finns det förvaringsutrymme för hjälpmedel såsom rullstol eller rullator tillgängligt för den boende i eller i anslutning till den egna lägenheten?	○	○	○
194. Finns det utrymme för de boende att vid behov förvara hjälpmedel nära vardagsrummet? (t ex rullstol, rullator)	○	○	○
195. Finns det förrådsutrymme där de boende kan förvara överblivna möbler eller säsongsutrustning?	○	○	○
196. Bidrar utrymmet utanför de enskilda lägenheterna till personlig utformning? (plats utanför dörren, alkov el liknande)	○	○	○
197. Ger inredningen karaktär av bostad och hem?	○	○	○

Trädgård/utomhusområde

198. Är utomhusmiljön avskärmad med staket eller buskage?	○	○	○

Trädgård/utomhusområde

199. Är grindar/staket utformade så att de skyddar de boende från att av misstag förirra sig ut?	◯	◯	◯
200. Finns det något område med doftande blommor?	◯	◯	◯
201. Finns det upphöjda rabatter?	◯	◯	◯
202. Kan trädgården/utomhusområdet användas av personer med funktionsnedsättningar? (ledstänger, ramper istället för trappsteg osv)	◯	◯	◯
203. Är alla ytor stabila och halkfria?	◯	◯	◯
204. Finns det någon gångslinga?	◯	◯	◯
205. Är trädgård/uteplats utformad så att den ger en karaktär av bostad och hem?	◯	◯	◯
206. Finns det plats i trädgård/utomhusområde för boende att ha ett eget område om de så önskar? (t ex egen rabatt, egna blomkrukor)	◯	◯	◯
207. Har alla boende möjlighet till utomhusvistelse via trädgård, uteplats eller balkong?	◯	◯	◯
208. Finns det valfrihet av flera trädgårdsområden? (t ex uteplats med sittplatser, gräsmatta, gångstig mellan landen)	◯	◯	◯
209. Finns det någon anläggning/hjälpmedel som underlättar för de boende kan hålla på med trädgårdssysslor? (t ex växthus, trädgårdsskjul, planteringslådor)	◯	◯	◯
210. Finns det separata sittplatser där man kan vara i fred?	◯	◯	◯
211. Är trädgård/utomhusområde skyddat från trafik- och industriljud?	◯	◯	◯
212. Är marken belagd med gräs eller grus?	◯	◯	◯

Trädgård/utomhusområde

213. Finns det sittplatser i trädgården eller utomhusområdet som kan användas av alla? (inkluderat personer med funktionsnedsättningar)	○	○	○
214. Kan man se aktiviteter i omgivningen? (trafikerade gator, affärer, skola)	○	○	○
215. Finns en variation av växter?	○	○	○

S-SCEAM – Nordin S, Elf M, McKee K, Wijk H.
